# Behavioral Repertoire Influences the Rate and Nature of Learning in Climbing: Implications for Individualized Learning Design in Preparation for Extreme Sports Participation

**DOI:** 10.3389/fpsyg.2018.00949

**Published:** 2018-06-12

**Authors:** Dominic Orth, Keith Davids, Jia-Yi Chow, Eric Brymer, Ludovic Seifert

**Affiliations:** ^1^Amsterdam Movement Sciences, Faculty of Behavioural and Movement Sciences, Vrije Universiteit, Amsterdam, Netherlands; ^2^Institute of Brain and Behaviour, Amsterdam, Netherlands; ^3^Centre for Sports Engineering Research, Sheffield Hallam University, Sheffield, United Kingdom; ^4^National Institute of Education, Nanyang Technological University, Singapore, Singapore; ^5^Institute of Sport, Physical Activity and Leisure, Leeds Beckett University, Leeds, United Kingdom; ^6^Faculty of Sport Sciences, Centre d'Etudes des Transformations des Activités Physiques et Sportives, CETAPS EA3832, University of Rouen Normandy, Rouen, France

**Keywords:** learning dynamics, scanning procedure, intrinsic dynamics, rock climbing, motor learning, system degeneracy

## Abstract

Extreme climbing where participants perform while knowing that a simple mistake could result in death requires a skill set normally acquired in non-extreme environments. In the ecological dynamics approach to perception and action, skill acquisition involves a process where the existing repertoire of behavioral capabilities (or coordination repertoire) of a learner are destabilized and re-organized through practice—this process can expand the individuals affordance boundaries allowing the individual to explore new environments. Change in coordination repertoire has been observed in bi-manual coordination and postural regulation tasks, where individuals begin practice using one mode of coordination before transitioning to another, more effective, coordination mode during practice. However, individuals may also improve through practice without qualitatively reorganizing movement system components—they do not find a new mode of coordination. To explain these individual differences during learning (i.e., whether or not a new action is discovered), a key candidate is the existing coordination repertoire present prior to practice. In this study, the learning dynamics of body configuration patterns organized with respect to an indoor climbing surface were observed and the existing repertoire of coordination evaluated prior to and after practice. Specifically, performance outcomes and movement patterns of eight beginners were observed across 42 trials of practice over a 7-week period. A pre- and post-test scanning procedure was used to determine existing patterns of movement coordination and the emergence of new movement patterns after the practice period. Data suggested the presence of different learning dynamics by examining trial-to-trial performance in terms of jerk (an indicator of climbing fluency), at the individual level of analysis. The different learning dynamics (identified qualitatively) included: continuous improvement, sudden improvement, and no improvement. Individuals showing sudden improvement appeared to develop a new movement pattern of coordination in terms of their capability to climb using new body-wall orientations, whereas those showing continuous improvement did not, they simply improved performance. The individual who did not improve in terms of jerk, improved in terms of distance climbed. We discuss implications for determining and predicting how individual differences can shape learning dynamics and interact with metastable learning design.

## Introduction

In recent years participation rates in extreme sports such as free solo climbing, where climbers perform in extreme environments without the use of safety aides such as ropes, have outstripped many traditional sports (Brymer and Schweitzer, 2013; Seifert et al., 2017). Performance in extreme climbing environments, where a fall would most likely result in death, places considerable physiological and psychological demands on the climber (Llewellyn et al., 2008). Deaths in climbing are most often attributed to climbing in extreme environments and when climbing without ropes (Lack et al., 2012). While the emotional and psychological requirements for climbing in extreme environments are often different from those required to climb in non-extreme environments, such as indoor climbing walls, many of the underlying skills required to complete a particular move when climbing a 3,000 m wall without ropes are the same as those required to undertake the same move in an indoor context (Brymer and Schweitzer, 2017).

Climbing in extreme environments not only requires a profound environmental knowledge and the ability to effectively assess environmental constraints such as weather conditions and rock or ice quality but also requires effective adaptability and highly tuned skills (Seifert et al., 2017). Understanding how individual climbers effectively acquire the skills required to perform at this level is important for the development of the individual climber and ultimately supports safer and more sustainable participation (Immonen et al., 2017).

### How environmental design can support adaptability in climbing

This study adopts the ecological dynamics approach to perception and action. Ecological dynamics integrates ideas from dynamical systems theory and ecological psychology toward understand learning and behavioral change by the individual toward becoming adapted to a particular environment (Rietveld and Kiverstein, 2014; Davids et al., 2015; Immonen et al., 2017). In this framework, behavioral change is underpinned by principles of self-organization which overtime, enhance the individual-environment fit (Schöner et al., 1992; Edelman and Gally, 2001; Sumpter, 2006). Self-organizing systems can be described as systems which are initially disordered and where global order can emerge under the influence of the system's own dynamics (Bruineberg and Rietveld, 2014, p. 4). The effort to satisfy current constraints (interacting environmental, task, and individual factors) gives rise to perceptual-motor couplings that function to support the individual's perception of affordances (opportunities) for action (Davids et al., 2008). By learning new ways of acting adaptive, or acting adaptive in new situations, the individual enhances their movement system degeneracy (i.e., the capability to use structurally different elements for the same functions: Kelso, 2012) which can extend the boundaries of what their environment affords for action (Orth et al., 2017c).

**Figure 1:**
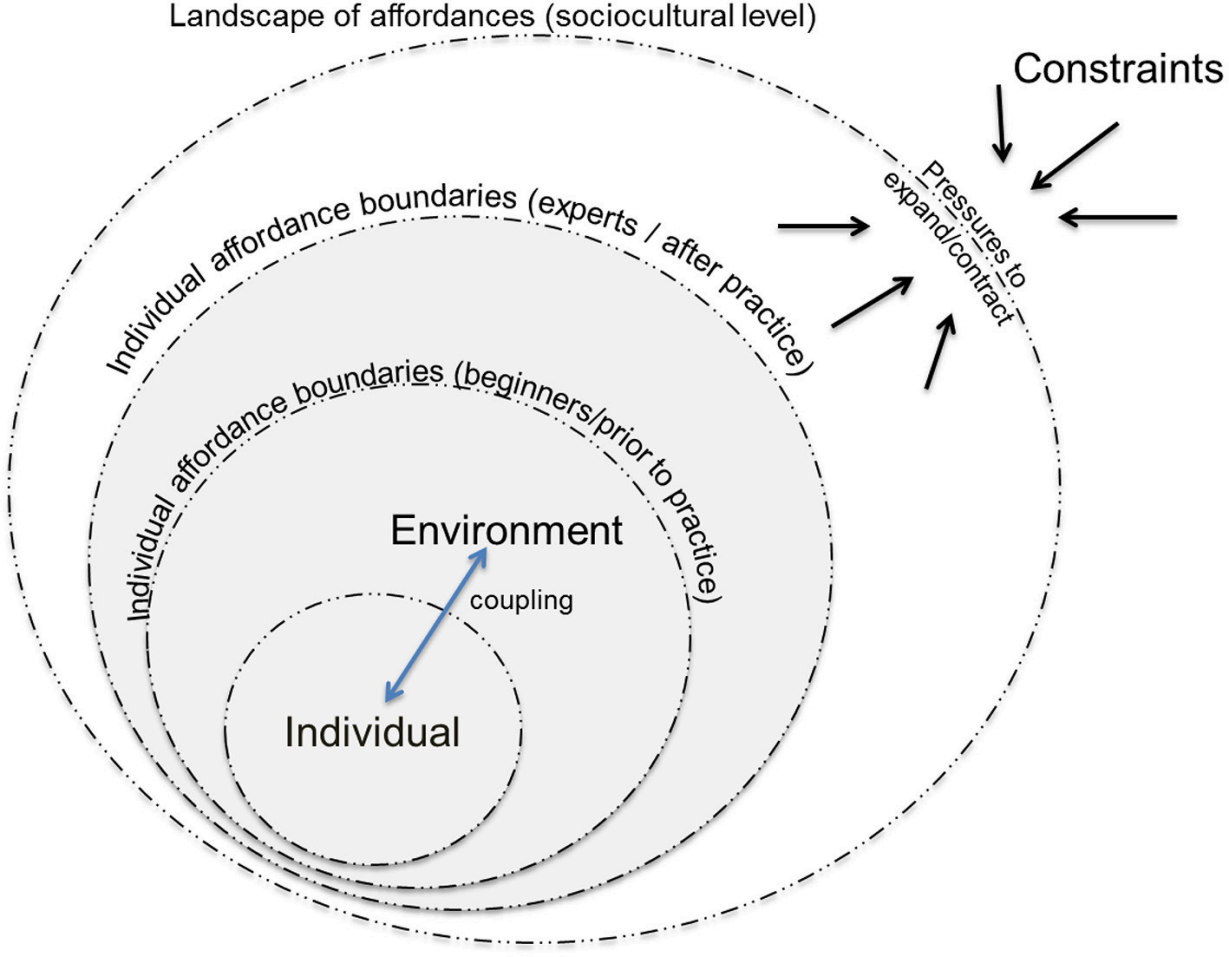
The individuals practice is nested within their learning and development. Constraints at both the individual and the sociocultural frame of reference, interact to impinge on perceptual-motor couplings, supporting an expansion or contraction of affordance boundaries. Through functional movement variability, including exploration, enhanced degeneracy, and discovery of new and functional actions the individual and social landscape of affordances can be enriched.

The idea of affordances, first introduced by Gibson (1979), suggests that successful behavior is predicated on the information-based relationship between the individual and their environment. The generation and pick-up of information supports the perception of affordances (or invitations for action) (Withagen et al., 2012). During practice, instability facilitates exploration of alternative motor solutions, and, hence their adaptability (Hristovski et al., 2011; Van Orden et al., 2011; Bril et al., 2012). Practically speaking, a coach or experimentalist might develop knowledge for affordances of other individuals (i.e., affordances the coach can provide by establishing a certain set of constraints). In doing so, the coach or researcher can interact with constraints so as to shape learning without prescribing or presupposing a solution in advance (Silva et al., 2013). Assuming that the individual behaves in such a way that takes into account the limits on their action capabilities (an hypothesis associated with affordance-based control: Fajen, 2007; Croft et al., 2018), when the individual is positioned at the limits of their affordance boundaries this can lead to an increase in movement variability during practice (Prieske et al., 2015; Orth et al., 2018), which may drive learning (Schöllhorn et al., 2009; Chow et al., 2011). For example, in climbing, the individual will control their actions in some respects based on how long they perceive they can continue to remain in contact with the wall, which can be influenced fatigue (Fryer et al., 2012). In order to manage fatigue, participants need to learn to use holds in different ways, such as with different grasping actions or with body positions that are more mechanically efficient. Through practice, as the individual becomes capable of using more efficient actions they will climb increasingly difficult routes or in more complex environments—reflecting their action boundaries, separating their ability to climb possible and impossible routes, have been expanded (Fajen, 2007). Figure 1 exemplifies these ideas, suggesting that constraints impinge on affordances at both the individual and the sociocultural frame of reference. Functional movement variability, including exploration, enhanced degeneracy, and discovery of new and functional actions are a behavioral mechanisms that support the expansion of affordance boundaries.

Operationalising these ideas, Orth et al. (2018) showed how a route designed with holds that could be grasped either using an overhand grip (like a ladder) or a side-grip (like grasping a cup handle) invited participants to carry out more exploratory actions during climbing (actions where individuals would touch a hold only to subsequently withdraw their hand to reposition it). Due to the route's design, beginners learned to explore holds whilst at the same time maintain climbing fluency when constraints were modified in a transfer test. In doing so, the learners extended their affordance boundaries (i.e., their ability to climb on new routes), by learning how to explore whilst maintaining fluency. According to Orth et al. (2018), the enhanced exploration in the dual-grasping route was because it allowed a fall-back option to an already stabilized movement pattern (grasping using the over hand grip and with body orientated face-on to the wall) thus, making exploration less risky. The study by Orth et al. (2018) also suggests that beginners need to learn how to use side-on body-wall orientations. Indeed, as shown by Seifert et al. (2015) intermediate skilled climbers tend to increase the amount of rolling motion at the hips when climbing holds were designed to promote these actions (specifically, edges running perpendicular to the ground plane were made available). Thus, the individual's current behavioral repertoire may have a strong influence on the nature and rate of learning in climbing tasks.

### The current study

The purpose of the current study was to determine to what extent the individual's current behavioral repertoire (i.e., the extant perceptual-motor landscape of performance solutions that can be adapted by the individual with respect to a particular environment: Davids et al., 2015) affect the learning dynamics and subsequent emergence of new skills. In order to determine if a solution is new relative to the individual, a scanning procedure can be used to assess an individual's current behavioral repertoire, allowing determination of how it changes during and after practice (Zanone and Kelso, 1992). For example, prior to learning a new skill, a scanning procedure can uncover pre-existing stable and unstable coordination solutions when performing under a given set of constraints. In doing so, this can be used to determine how the perceptual-motor landscape is altered by practice (Zanone and Kelso, 1992). The main aim of this study was to evaluate, in beginner climbers, possible relationships between learning dynamics and the emergence of new patterns of movement coordination during skills practice in a route climbing task. We also aimed to evaluate, using a scanning procedure, any mediating relationship between the learning dynamics and the learners behavioral repertoire as assessed prior to and after practice.

In order to assess learning, we used variables that quantify climbing fluency (Orth et al., 2017b). Fluency in climbing is globally captured as jerk (a measure sensitive to the number of sub-movements made while climbing: Seifert et al., 2014), and can further be assessed separately along temporal and spatial dimensions (Orth et al., 2017b). To assess temporal performance, mobility is used and reflects the time spent moving relative to remaining stationary (Billat et al., 1995). To assess spatial performance, the entropy of the hip trajectory is used, where more straight forward trajectories are associated with behavioral certainty (Cordier et al., 1994b).

Using these variables, performance was observed over an extended time period (7 weeks of practice, 2 sessions per week). During practice we encouraged exploration by designing a climbing route where each hold had four good edges (top, bottom, and sides). The route also encouraged exploration of different pathways though the route. Following previous results (Seifert et al., 2015; Orth et al., 2018), we anticipated beginner climbers would be able to immediately climb the route with a face-on orientation to the wall. However, in order for beginners to substantially improve their climbing fluency, they would need to also use side-on body wall configurations. We predicted that the discovery of these new movement patterns would support a sudden improvement in performance. Additionally, we expected these movement patterns to absent prior to practice and be present after practice.

## Methods

### Participants

Eight participants without prior experience of outdoor rock climbing were recruited to be involved in the learning study, noting that one dropped out during the experiment (see Table 1). Noting however, that participants had received a minimum of 10h of practice in indoor climbing, because this amount of practice was the minimum required to guarantee that participants can correctly know to set harness, rope and know belaying. Moreover, participants had roughly a 16–18 Ewbank skill level and so were not completely inexperienced. Further inclusion criteria required that participants be within the healthy BMI range (<25) and have an arm span of no <140 cm. This was done to ensure that climbers were able to reach holds as intended by two professional route setters. Notably one participant (P14) had a BMI of 26.8. However, because this result was because the individual had a larger proportion of muscle mass, he was permitted to participate. All participants were right handed.

**Table 1:** Participant details.

**Learners**	**P12**	**P13**	**P14**	**P15**	P17^**^	**P18**	**P19**	**P21**
Age (years)	18.0	19.0	21.0	20.0	21.0	22.0	24.0	18.0
Gender	F	M	F	M	F	F	M	F
On-sight ability (Ewbank^*^)	17	17	17	18	17	17	18	16
Standing height (cm)	162	182	186	171	176	156	165	163
Arm span (cm)	162	185	173	178	174	152	166	166
Body weight (kg)	54.6	68.4	83.0	58.5	64.2	53.0	72.5	59
Grip strength (kg)	26.2	54.6	52.0	26.0	28.3	23.8	58.0	22.5
Grip strength to weight ratio	0.48	0.80	0.63	0.44	0.44	0.45	0.88	0.38

* *For conversions from Ewbank across other systems see Draper et al. (2011a)*.

** *Dropped out during intervention*.

Finally, testing occurred as part of a physical activity course for students enrolled at local university (Rouen Normandy University). In participating, they received a grade for their participation for the climb course unit. The local ethics committee of the Rouen Normandy University approved the protocol, who verified that wearing data acquisition equipment was compatible with climbing, which was validated. This study was carried out in accordance with the recommendations of the guidelines of the International Committee of Medical Journal Editors. The protocol was explained to all participants who gave written informed consent in accordance with the Declaration of Helsinki.

### Experimental design and climbing route design

**Figure 2:**
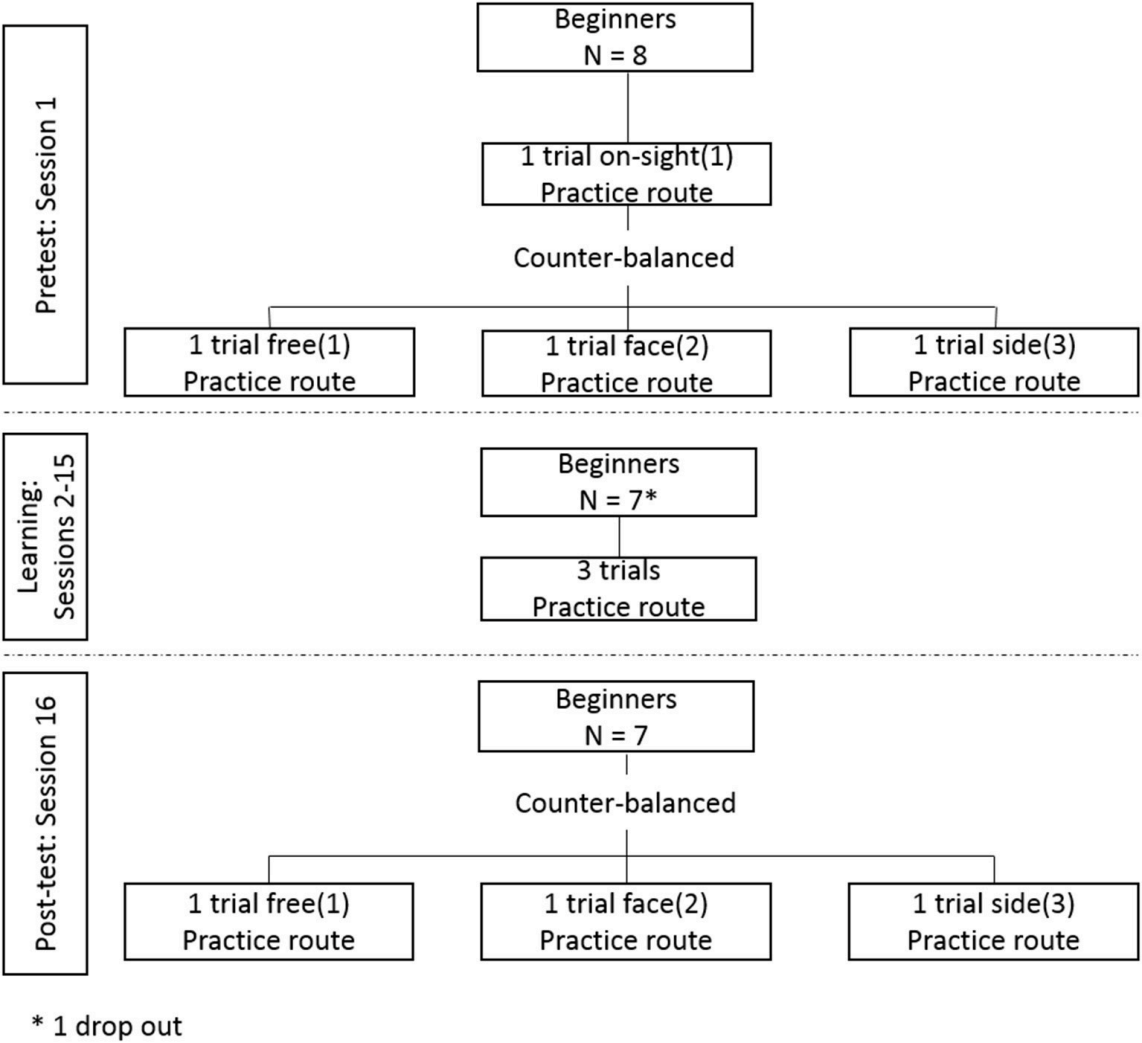
Experimental design.

The study involved two pre-test and two post-test sessions, and 14 learning sessions in total. The learning sessions were distributed such that two learning sessions per week (e.g., Tuesday, Friday) were carried out over a 7-week period. Within each session participants were required to climb the same route three times, equating to 42 trials of practice overall. The volume of practice corresponded roughly to the typical length of a beginner level climbing course and matched values in existing studies reporting the acquisition dynamics of multi-articular skill (Delignières et al., 1998; Chow et al., 2008a). Pre-testing was carried out 1 week prior to commencing the learning sessions and post-testing was carried out 1 week after the final learning session (see Figure 2).

Across all testing sessions, participants upon arrival were fitted with climbing shoes and afforded a 10-min period to warm up their hands, feet, and body. Aside from stretching and mobility, during this time a very easy traverse was also carried out to allow participants to safely use their fingers and were prepared to support their body weight. They were then fitted with a harness and instrumentation (detailed below). Prior to undertaking each climb, the following global task instructions were given: “climb the route as fluently as possible, minimizing jerky movement, taking an efficient path through the route and minimizing prolonged pauses.” Participants were then afforded a 3-min period to view the route from the ground, following which the trial was commenced. Between each climb, a seated 5-min rest was enforced to minimize effects of fatigue on performance. Globally, all routes were designed at 5b F-RSD by agreement of two qualified route-setters (Draper et al., 2011b). This difficulty was chosen as it corresponds to a beginner level of difficulty (Draper et al., 2011a).

### Pre- and post-test scanning procedure

#### Session 1 and session 16

The pre- and post-test sessions required participants to undergo a scanning procedure modified to the climbing task and required three performance trials carried out on the same day. In theory the approach involves scaling a parameter, in this case required body position, and observing effects on overall movement coordination and climbing fluency (including jerk, mobility, and entropy).

**Figure 3:**
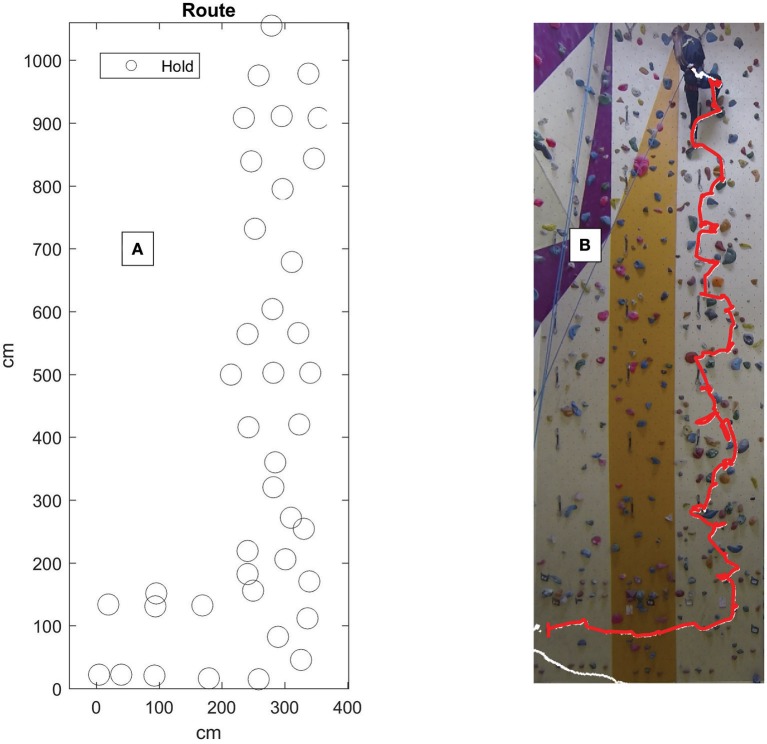
Route design. **(A)** Circles indicate the location of the holds of the route used for the scanning and learning procedure. **(B)** Shows an example of the hip tracked and mapped over a photo of the route. The redline indicates the hip tracked from the beginning the end of the climb.

In order to assess how beginners organized their body with respect to the wall while climbing the orientation of the pelvis with respect to the wall was gathered while mobile. Obtaining the pelvis orientation during mobility was important as we were interested in body-wall coordination modes during route progression and not during resting (see the example given in Figure 3). Thus, the scanning procedure was designed to assess participants' capacity to coordinate different body-wall positions while mobile (when displacement at the hips was occurring).

The first climbing condition of the scanning procedure, acted as a reference and required individuals to climb under global task instructions (i.e., to get to the end of the route). The second condition required participants to climb the same route under instructions to maintain as much as possible the front of their body facing the wall (i.e., the “face-on” condition). The third condition required climbing with the side of the body facing the wall for as much as possible (i.e., the “side-on” condition). It was anticipated that the beginners would show better performance under the face-on condition, but, be unable to remain mobile when in the side-on condition. The order for each condition was counterbalanced to control for possible order of treatment effects. Participants were not given time to preview the route for the scanning procedure. Note also that the scanning procedure was carried out on the same route as the learning route. Figure 3A shows the position of each hold of the scanning and learning route with respect to the climbing wall plane.

### Learning sessions: route and procedures

#### Session 2 to session 15

Learning sessions were carried out twice weekly with at least 2 days in between (e.g., Tuesday, Friday) and over a 7-week period. Within each learning session participants carried out three trials of practice per session on the same route. Between trials, participants were required to sit for no less than 5 min between trials without viewing the route. Before each trial they were allowed to preview the route for a maximum of 3 min if desired. Before participants climbed for the first time they were informed that they would be given feedback about their performance in terms of jerk, entropy and immobility. These data were also explained to participants in terms of how movements at the hips affects these values. Jerk was explained to increase the more they fluctuated between increasing and decreasing their speed while climbing. Entropy was explained to increase with the more movements they used to get to the top. Immobility was explained to increase the longer they stayed still. Finally, participants were informed lower values of jerk, entropy, and immobility were indications of better performance. All instructions, belaying, and feedback were given by the same researcher across all sessions.

At the beginning of each learning session (not including the first session) feedback of climbing fluency was provided regarding the previous learning session. In addition to this, participants were emailed their feedback 48 h after each learning session. Specifically, the feedback given included three values, jerk, entropy, and immobility and also the adopted trajectory through the route for each trial. The climbed trajectory was conveyed in the form of photo overlay of their climbed trajectory onto a photo of the climbing wall. Figure 3B is an example image given to participants that exemplifies the data given.

### Instrumentation

**Figure 4:**
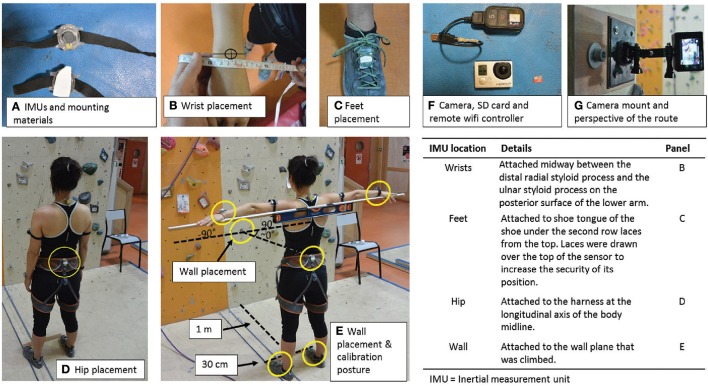
Instrumentation. **(A–E)** show the inertial measurement unit placement and initial calibration posture (see embedded table, bottom right, for details). **(F,G)** show the instrumentation and positioning of the camera used to record each climb.

Data on directions of the trunk (3D unit vectors in Earth reference) were collected from small, wearable, inertial measurement units (IMU: Figure 4A). These IMUs contain three sensor components: a tri-axial accelerometer (±8G); tri-axial gyroscope (1,600°/s); and a tri-axial magnetometer (MotionPod, Movea©, Grenoble, France). Data collected from the IMUs were recorded with North magnetic reference at 100 Hz and transmitted by wireless connection with a control unit run off a desktop operating system. IMUs were attached to the hip to estimate the movement at the climber's center of mass without interfering with movement (Figures 4B–D). Finally, in order to orientate the sensor with respect to the vertical and wall references, participants were required to adopt a calibration position prior to each climb. This position was recorded for 10 s prior to each attempt (Figure 4E) and the average value was used to zero the angular positions relative to the wall reference. The same sensor and relative placement location, orientation, and procedure were used throughout the entire experiment.

Raw sensor recordings were then prepared. Specifically, gyroscope, accelerometer, and magnetometer information were converted into a 3 × 3 rotation matrix that describes each sensor in an Earth frame (North, West and vertical). Specifically, as described in, Boulanger et al. (2016) two of the sensor components (accelerometer and gyroscope) in a rotation signal provides a better signal/noise ratio and the third sensor, the magnetometer, is used to obtain the Earth reference. A transformation is then performed through a complementary filter based algorithm (as described in, Madgwick et al., 2011).

Each trial was also captured with a frontal camera (GoPro© Hero 3) fixed 9.5 m away from the climbing wall and at a distance of 5.4 m from the ground and operated via remote wi-fi, with recordings directly captured to a SD card (Figures 4F,G). A red light was equipped to the back of the harness midpoint. Post processing involved automatic tracking of the red light position on a frame by frame basis (Boulanger et al., 2016). In order to synchronize the signals, we used the obtained trajectory data from the video to compute a time series estimating the acceleration of the pelvis. Then, using a maximum correlation measure between the sensor-recorded accelerations of the pelvis (the norm of the lateral and vertical components), the delay between each signal, video and sensor, was estimated (Boulanger et al., 2016). Finally, the start (first discernible reaching action made from quadrupedal support) and end (first discernible contact made with the final hold with both hands) were then manually determined from the video and the two signals extracted over the same time period accounting for the delay obtained in the previous step.

### Computations

From the raw signals recorded during each climb, three forms of data were computed to assess performance and learning dynamics:

- jerk coefficient of translation–jerk reflects the spatial-temporal indicator of performance;
- geometric index of entropy–entropy reflects the spatial indicator of performance, and;
- the threshold based immobility to mobility ratio–immobility is the temporal indicator of performance.

Finally, in order to assess the body state dynamics, we took the orientation of the hip-wall angle when the individual was mobile.

#### Jerk

Jerk (the derivative of acceleration) is correlated with the number of sub-movements that compose gross actions (Seifert et al., 2014). The fewer sub movements made, the lower the jerk value.

When the trajectory is known, such as when the hip position is tracked relative to the wall surface, allowing that for a given trajectory *x*:[*O, T*] → *R*^3^, the dimensionless jerk coefficient of translation is defined as:

(1) $$\begin{array}{l} Jerk=\;\frac{T^{5}}{{\left(\Delta x\right)}^{2}}\;\int_{0}^{T}||\frac{d^{3}x}{dt^{3}}\left(s\right)|{|}^{2}ds \end{array}$$

Noting that Δ*x* is the length of the climbed trajectory.

#### Geometric index of entropy

The geometric index of entropy is a ratio of the path length of a trajectory to the perimeter of its convex hull and is a uniquely spatial indicator of performance (Cordier et al., 1994b). For a given trajectory *x*:[*O, T*] → *R*^3^, letting Δ*x* as the distance of the path covered by the hips, and Δ*c* the perimeter of the convex hull, we find:

(2) $$\begin{array}{l} Entropy_{x}=\;\frac{\log\;\left(2*\Delta x\right)-\log\left(\Delta c\left(x\right)\right)}{\log\;\left(2\right)} \end{array}$$

Note that the division by log(2) places the geometric index of entropy in dimensionless terms (bits). Thus, the greater amount of displacement that occurs within a given convex hull, the higher this value and more complex (or chaotic) the movement trajectory.

#### Threshold based immobility to mobility ratio

The relationship between periods of mobility to immobility is estimated by determining how long, with respect to the total climb time, an individual's COM remains in a stationary state, relative to a moving state. It is a uniquely temporal indicator of performance (Orth et al., 2017b). Time spent “immobile” reflects time under isometric contraction, incurring an energy cost (Billat et al., 1995). Since hip mobility is determined as a given level of displacement over time, a solution to remove potential operator bias is to directly use hip velocity, and apply a threshold. Thus, for this study a threshold value was applied to the velocity of the climber's trajectory.

Specifically, for a trajectory *x*:[*O, T*] → *R*^3^, we find the threshold based immobility to mobility ratio as:

(3) $$\begin{array}{l} {Ratio\;of\;immobility\;to\;mobilty}_{x}=\frac{\sum_{i=1}^{N}P_{i}}{N} \end{array}$$

(4) $$\begin{array}{l} P_{i}=\{\begin{array}{l} 1,\;\\ \;if\;v_{i}<threshold\\ 0,\;\\ \;if\;v_{i}\geq threshold \end{array} \end{array}$$

(5) $$\begin{array}{l} v_{i}=f\sqrt{x_{i}^{2}+y_{i}^{2}} \end{array}$$

Hence the larger the threshold based immobility to mobility ratio, the longer the individual's respective COM is considered to be in a more immobile state.

#### Orientation of the trunk when mobile

**Figure 5:**
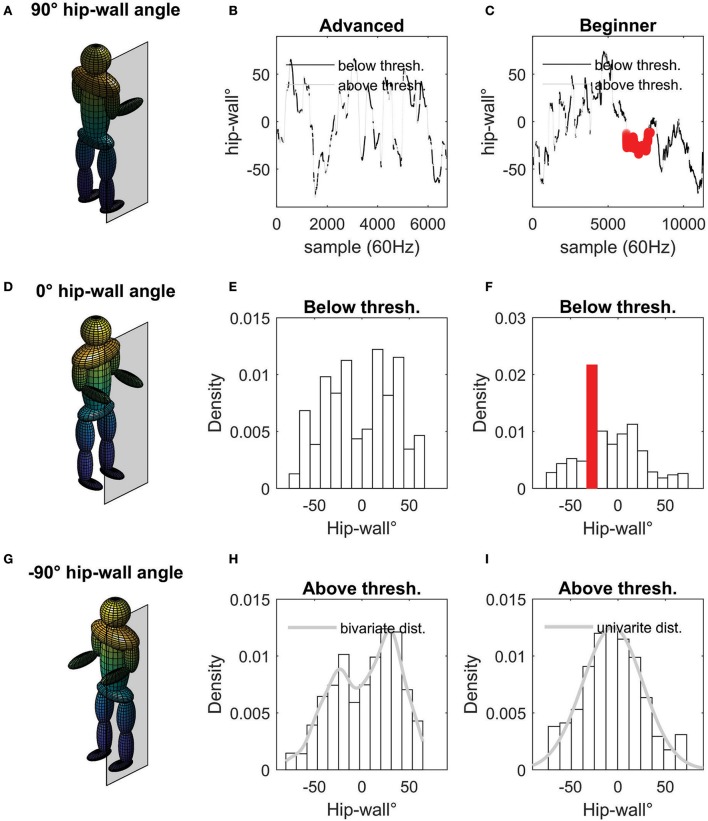
The hip-to-wall angle data reduction. The mannequins **(A,D,G)** on the left show two general coordination patterns: face-on (middle mannequin), and; side-on (top and bottom mannequins). The middle column of graphs **(B,E,H)** shows the angular position of the hip of an expert climber. The time series data of the hip **(B)** shows the hip-wall angle when moving below 20 cm/s (black line) and above (gray line). When moving below 20 cm/s the climber is considered “immobile” (**E** shows these data as a frequency histogram). When moving above 20 cm/s the climber is considered “mobile” **(H)**. For contrast, a beginners data is shown **(C,F,I)**. When the beginner is immobile they may rest in a side-on position **(F)**. However, beginners find it difficult to use a side-on body-wall position when mobile **(I)**. Note that density (y-axis) of the histograms is used in place of frequency as these data have been normalized so that the area under the curve is equal to one. dist. = distribution. thresh. = threshold.

The hip-wall orientation was taken as the angle formed between the hip sensor and a sensor positioned on the climbing surface (recall Figure 4E). Following Seifert et al. (2015), the time series of rotation around the axis perpendicular to the transverse plane was extracted using the wall reference such that 0°Corresponded to a face-wall position (the sagittal plane perpendicular to the climbing surface; and following the right hand rule, 90° rotation right side of the body is parallel to the wall plane, and; −90° left side of the body is parallel to the wall plane (Figure 5A). It was anticipated that when participants were requested to climb side-on to the wall, the body-wall angle distributions during mobility would be concentrated around 0° in the pre-test, and, more toward ±90° in the post-test. Thus, the probability distributions of the hip-wall angle segmented above threshold were used to assess the initial coordination and changes after practice revealed when undergoing the scanning procedure.

Indeed, it was found in pilot work that adopting a side-wall orientation could be achieved by experts while using holds for route progression. This is shown in Figures 5D–F. In contrast, beginners were less capable of using the side-on position when mobile. However, during periods of immobility it was anticipated that beginners might rest in the side-on position (as shown in Figures 5D, 4E). As described in Fuss et al. (2013), skill effects related to more advanced movement patterns can require that the climber achieve a threshold of mobility in conjunction to the relative positioning of the COM with respect to wall. Thus, it was important to segment the hip-wall orientation according to states of more or less mobility. To separate between states of mobility and immobility, we chose to set a threshold of movement at the hip of 20 cm/s which allowed a feasible and objective quantification of mobility. Whilst previous work has classified an individual as immobile using frame-by-frame analysis of an operator, this is extremely time consuming and open to operator bias. For example, criteria for mobility have included statements like: “progress of the hips was observed” (Billat et al., 1995) whereas, criteria for static climbing have included: “no discernible movement in pelvic girdle” (White and Olsen, 2010). Figure 5, provides face validity of using 20 cm/s, shown in the normalized density histograms comparing the hip-wall angles segmented above and below the threshold used to determine mobility (for additional discussion see: Orth et al., 2017b).

### Statistical procedures

The experimental design for addressing pre- and post-test findings required a repeated measures ANOVA with three levels of instruction (free, face, and side) and two levels of time (pre and post). In cases where main or interaction effects were significant, planned contrasts were carried out assuming that variables indicating skilfulness (i.e., jerk, entropy, and immobility) would improve due to practice. For effects related to condition, it was anticipated that performance in the side-on condition would be worse relative to the face-on condition (i.e., revealed in higher levels of jerk, entropy and degree of immobility relative to the face-on condition).

To examine learning dynamics, a one-way repeated measures ANOVA was used with planned contrasts to assess, at the group level, at what point a plateau in performance was evident by contrasting each trial with the final trial. It was anticipated, that the level of mobility would plateau after entropy (Cordier et al., 1994b, 1996; Orth et al., 2017a).

When the sphericity assumption was violated in the repeated measures variables, Greenhouse-Geisser adjustments are made. Finally, effect sizes, were reported in cases where a focused effect is addressed (i.e., comparisons involving two groups) by converting *F*-ratios to *r*-values following Field (2009, p. 501). Noting that: *r* = 0.10 reflects small effect; *r* = 0.30 is a medium effect, and; *r* = 0.50 is a large effect. Additional follow-up tests beyond planned contrasts were done using pair-wise (dependent) *t*-tests with Bonferroni corrections. All statistics were run using IBM® SPSS® Statistics version 21. All effects are reported at a statistical significance level of *p* < 0.05.

## Results

In the following sections, group outcomes of the pre- and post-test on the scanning procedure are given. We then address the learning dynamics, finally moving to individual analyses. Note that since participant 17 did not complete the practice intervention, she was removed from any statistical analysis.

### Results at the group level

#### Grouped pre- and post-test scanning procedure

**Figure 6:**
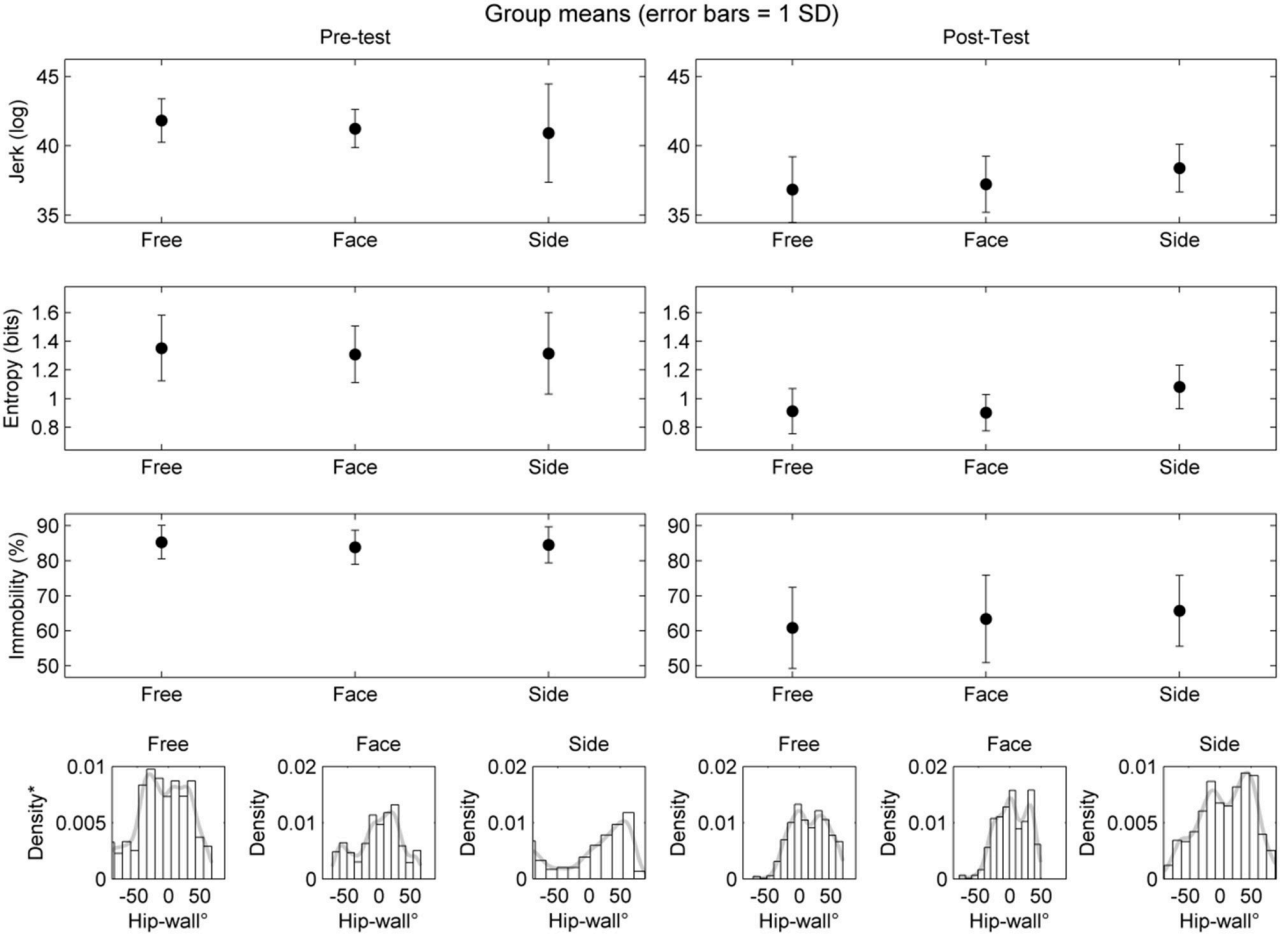
Grouped outcomes from the scanning procedure for the pre-test **(left)** and post-test **(right)**. Only the main effect of practice was found to be statistically significant. Note that free refers to the condition where climbers climbed as they liked. Face refers to the condition where climbers were asked to try to climb the route while maintaining as much as possible facing the wall with the front of their body. Side refers to when climbers were asked to climb the route as much as possible with the side of their body facing the wall. ^*^ = y-axis of histograms represent the normalized density of the hip-wall angle during mobility.

The outcomes, jerk, entropy, and degree of mobility of the scanning procedure were assessed across three levels of instruction (free, face, and side) and two levels of time (pre and post). There was a significant main effect of practice on: jerk, *F*_(1, 6)_ = 11.56, *p* < 0.01, *r* = 0.81; entropy, *F*_(1, 6)_ = 72.96, *p* < 0.001, *r* = 0.96, and; level of mobility, *F*_(1, 6)_ = 59.53, *p* < 0.001, *r* = 0.95. The decreases in jerk, entropy, and immobility after practice were all large effects. There were no significant effects for instruction, nor was there a significant interaction between practice and instruction. Also shown in Figure 6 are the hip-wall angle distributions (bottom row of histograms). These findings suggest that, at the group level, participants were capable of being mobile and oriented face-on and side-on to the wall both before and after practice.

#### Grouped learning dynamics

**Figure 7:**
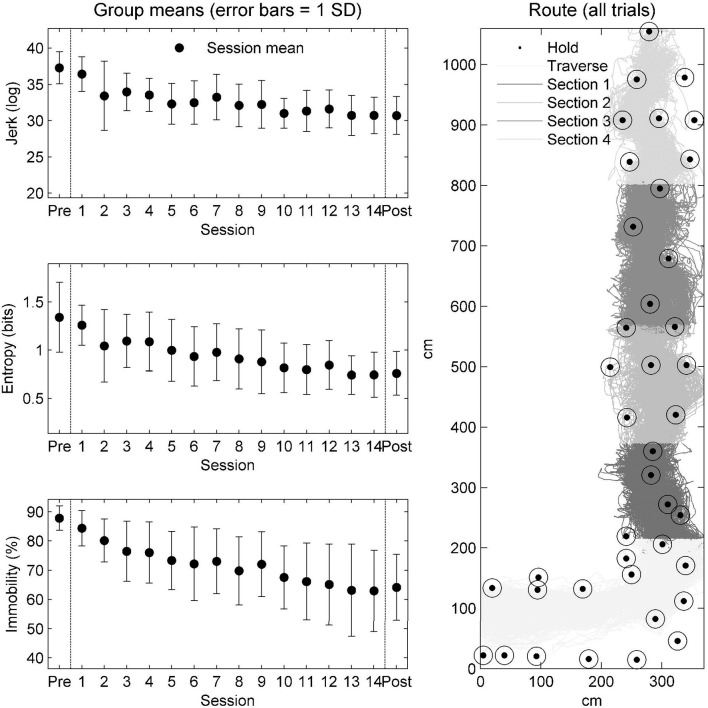
Grouped outcomes through practice. The graphs on the left show the session average and standard deviations of jerk **(top)**, entropy **(middle)**, and immobility **(bottom)** for the seven climbers followed through practice. The graph on the right indicates the design characteristics of the route. Dotted circles indicate the position of the holds. The hip position of all trials of practice across all participants onto the wall plane. The different shades of the hip data are a function of the section of the route (see the legend).

The grouped outcomes showed that across each outcome (jerk, entropy, and immobility), an improvement in performance was observed through practice (see Figure 7 which summarizes the session average for each variable). The main effect of trial was significant for all outcomes: jerk, *F*_(1, 13)_ = 5.15, *p* < 0.001; entropy, *F*_(1, 13)_ = 4.68, *p* < 0.001, and; level of mobility, *F*_(1, 13)_ = 11.62, *p* < 0.001.

Repeated contrasts were then performed to evaluate whether performance on each outcome variable improved at the same or different rates. Contrasts were, therefore, set up to compare each session relative to the penultimate session of practice (session 14). When contrasts were not statistically significant to the final trial of practice, performance can be considered plateaued (Cordier et al., 1994b). These outcomes are summarized in Table 2, which shows that both jerk and entropy values plateaued at session 7 whilst participants continued to improve their level of mobility until session 9.

**Table 2:**
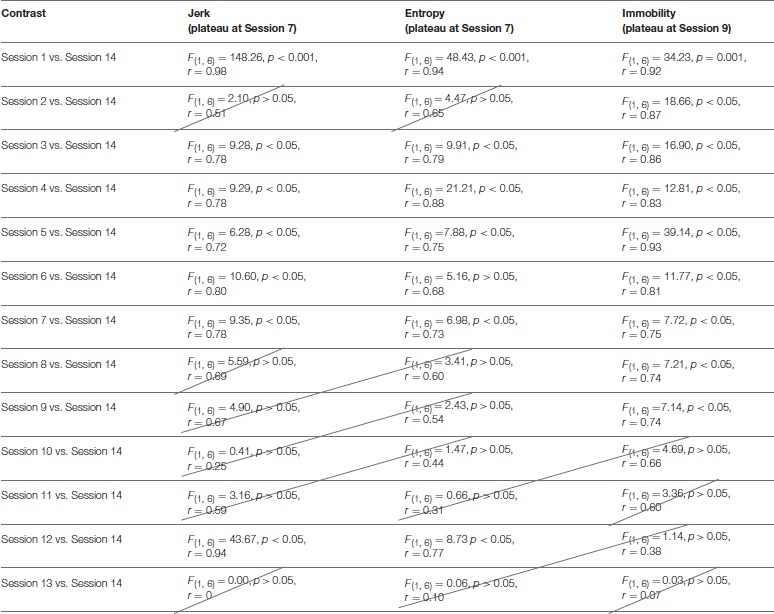
Group level (*n* = 7) contrasts of each trial against the final trial for each outcome variable.

*For mean differences and directions of effects see Figure 7*.

*Note that diagonal lines indicate that the differences in the outcome variable during climbing between the final session of practice and the first session of practice were not statistically significant (alpha set at p < 0.05)*.

We then performed a final follow-up test to address whether any sudden improvements in performance could be identified at the group level. That is, the purpose of the follow-up tests were to examine whether from one session to the next if, at the group level, performance could be shown to improve gradually or abruptly. Pairwise (dependent) comparisons were performed comparing each session of practice to the next session (e.g., session 1 vs. session 2, session 2 vs. session 3, and so on until session 13 vs. session 14). These were performed (with Bonferroni corrections) on jerk, entropy, and immobility values. No statistically significant differences between sessions were uncovered.

## Discussion

### Grouped outcomes

At the group level, findings are generally in support of previous literature in climbing. Jerk, entropy, and immobility have all been implicated as indicators of skill in climbing (Cordier et al., 1994b; Billat et al., 1995; Seifert et al., 2014), and is well corroborated in these data, each showing clear tendencies to improve through practice. This is the first study that examined jerk, entropy, and immobility in combination and we anticipated that participants would learn to co-vary movement complexity (entropy) with climbing mobility (Orth et al., 2017b), but that, initially, these two outcomes would improve at different rates through practice (Cordier et al., 1994a, 1996; Orth et al., 2017a). The latter expectation is supported, the former is not.

In this first instance, we expected that the learners would increase movement complexity and level of mobility in the side-on condition. In doing so, this should help to maintain a stable level of jerk (Orth et al., 2017b). The outcomes, when compared across the pre- and post-test, did not support this prediction–in so far that they did not reveal a significant interaction of route and time (pre- vs. post-test) for any of the outcome variables. A reason for this may be that the post-test scanning procedure was carried out on the same route as was practiced. This probably led to a tendency to climb faster compared to unfamiliar routes, and without needing to adapt movement complexity alongside mobility when using either the face-on or side-on body positions.

Additionally, at the group level, we did not find a clear indication that prior to practice, the beginners needed to learn how to climb, whilst mobile, in a side-on position. As shown in the probability density plots in Figure 6, these findings suggest that, as a group, the individuals had the capability to immediately adapt this position–and that further practice was beneficial at a level of general refinement or optimization of movement parameters supporting fluent climbing (Chow et al., 2008b; Hristovski et al., 2011).

These ideas are generally supported in the data on learning dynamics at the group level (see Figure 7), where over practice, improvement in terms of jerk, entropy, and immobility followed a fairly linear progression (also generally corroborated with the lack of significant session to session differences tested in the follow-up). However, given the large standard deviations present at the group level, additional individualized analyses were carried out. Indeed, examining the grouped hip tracings in Figure 7 shows that a large range of climbed trajectories were used and prompted us to carry out an exploratory analysis to examine any important differences at the individual level (Liu et al., 2006).

### Individual analysis: qualitative assessment of the learning curves

#### Individual learning dynamics and their relationship to the scanning procedure

**Figure 8:**
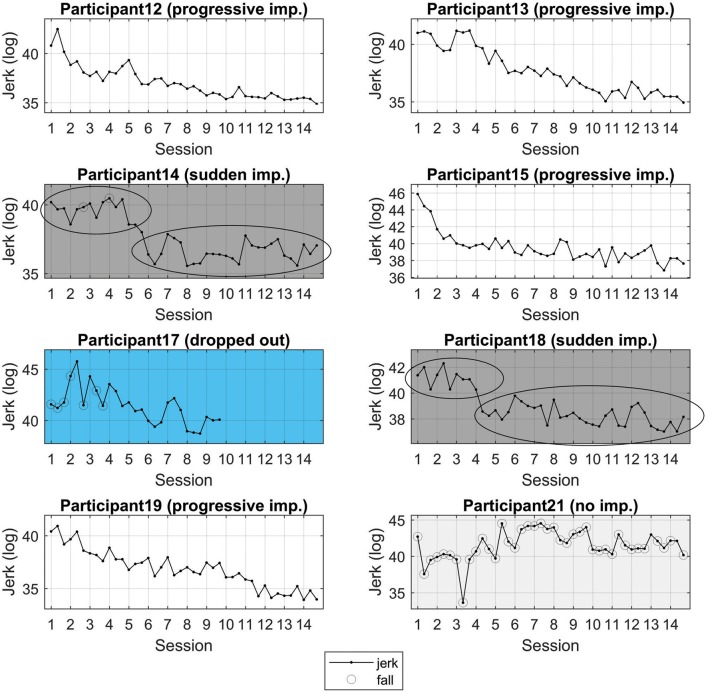
Individual learning curves. Three types of learning curves are apparent. Continuous improvement (participants 12, 13, 15, and 19); sudden improvement (participants 14 and 18), and; no improvement (participants 21). imp. = improvement. Note that here different scales are used in the y-axis in order to accentuate the nature of the learning curve for each individual. Different color schemes are used to highlight subgroupings.

In the first step of the exploratory analysis, we examined each of the individual's learning curves. Here we present the values on jerk for each trial of practice (jerk is presented since is a spatial-temporal indicator of fluency and provides a more global indication of change than immobility and entropy; Orth et al., 2017b). In doing so, three types of curves were qualitatively identified (see Figure 8):

- progressive (continuous) improvement (participants 12, 13, 15, and 19);
- sudden improvement (participants 14 and 18 and possibly 17);
- no improvement (participant 21).

**Figure 9:**
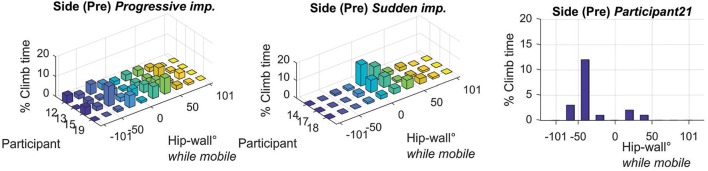
Histograms of each individual's hip to wall angle, expressed as a percentage of time spent climbing under the side-on condition during the pre-test. The left graph shows the data for the participants 12, 13, 15, and 19. Note the spread out nature of the hip-wall angle data. The middle graph shows the data for the participants 14, 17, and 18. Note the concentrated nature of the hip-wall angle. The right graph shows participant 21. The sub groups were formed according to the nature of the learning curves discussed and shown in Figure 8. Note that, based on the histogram data here, participant 17 was included with the sudden improvement group as her data is highly concentrated around 0 degrees.

After identifying these differences using the individual learning curves, we re-examined the pre- and post-test hip-wall angle data grouped as progressive; sudden improvement, and; no improvement. These data, presented in Figure 9, suggest that the initial capability to climb side-on to the wall while mobile affected the learning dynamics. Specifically, the most compelling findings as shown in Figure 9, which suggests that for participants where the hip wall angle was well spread from −90 to 90 degrees in the pre-test showed a progressive improvement during learning. For participants who showed a concentration of the hip-wall angle around 0 degrees in the pre-test showed a sudden improvement during learning.

#### Progressive improvement

**Figure 10:**
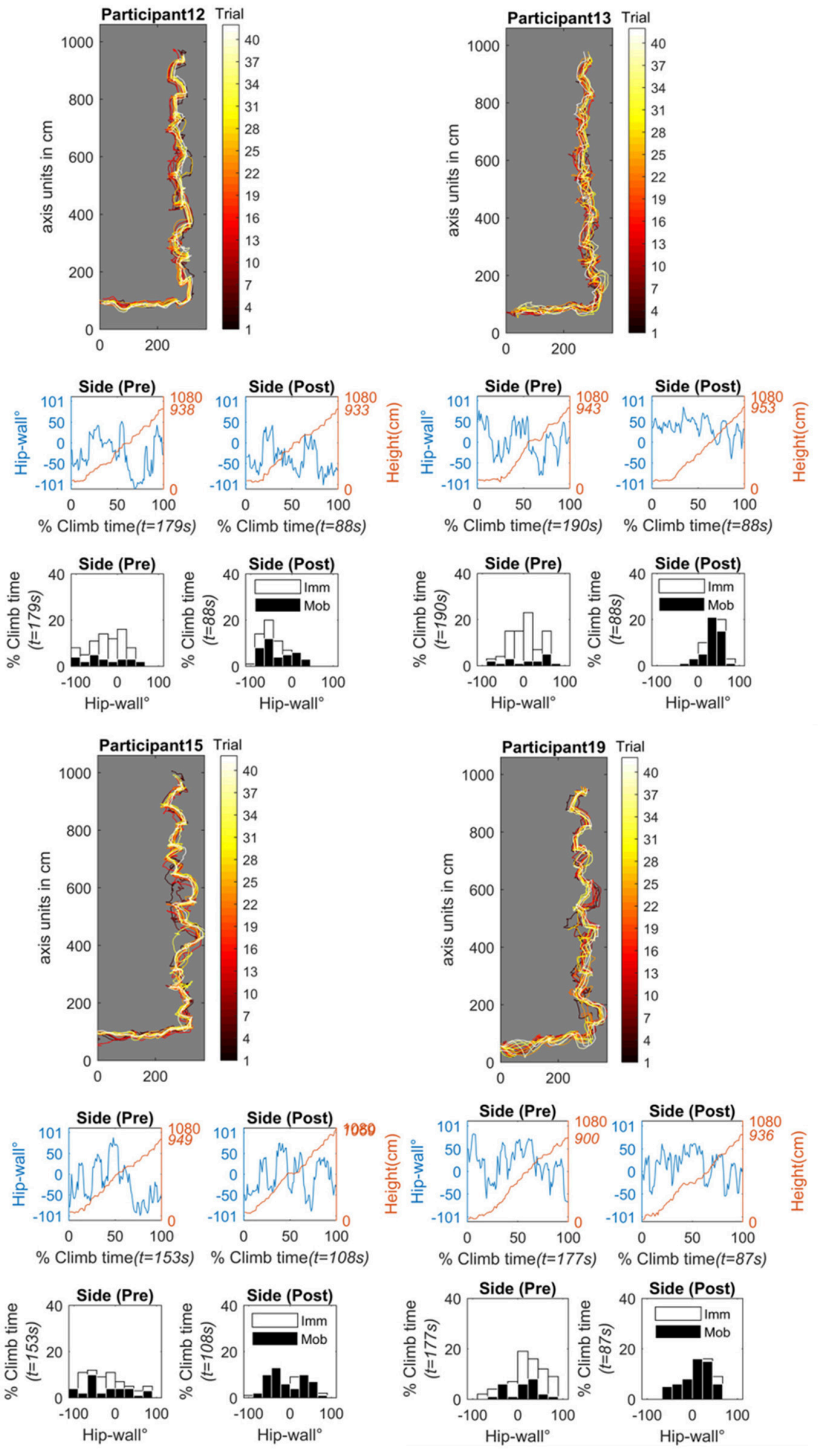
Progressive improvement subgroup made up of participants 12 **(top–left)**, 13 **(top–right)**, 15 **(bottom left)**, and 19 **(bottom right)**. For each participant, all practice trials are projected onto the wall plane (the black trace represents trail 1, the white trace is trial 42). The time-series graphs show the pre-test and post-test of the side-on condition. Shown are the time-normalized hip-wall angle (primary y-axis, blue line) and the time-series of the height of the hip position (secondary y-axis, red line). The histogram graphs show the pre- and post-test of the side-on condition. Shown is the relative time spent in different body-wall angles while mobile (black bins) or immobile (white bins) angle in degrees. Imm, Immobile; Mob, Mobile; t, total time.

Participants who appeared to improve progressively during practice, in the pre-test when asked to climb as much as possible with the side of their body relative to the wall, this hip-wall angle reflects a flatter, more spread distribution (see also the pre-test post-test histograms for each individual in Figure 10). By examining the time normalized raw data of the hip roll (see the primary axis of the line plots for each individual in Figure 10), it appears these individuals were able to transition multiple times from around 0° (indicating a face-on position) to more oblique angles toward positive or negative 90°.

#### Sudden improvement

**Figure 11:**
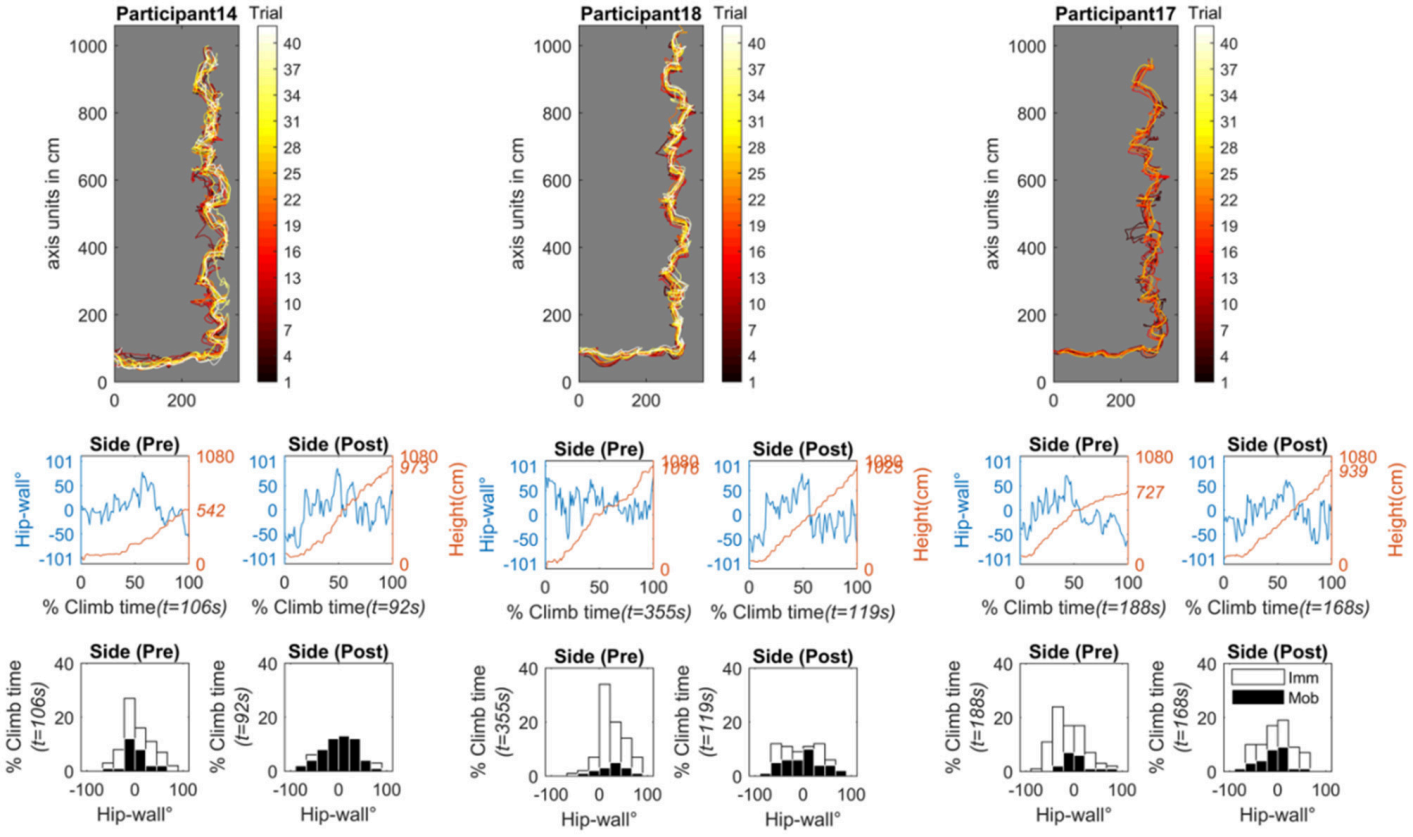
Sudden improvement subgroup made up of participants 14 **(left)**, 18 **(middle)**, and 17 **(right)**. Note participant 17 (the participant who dropped out after practicing 27 trials) was also added to this subgroup after examining the participant's histogram results relative to participants 14 and 18 (recall Figure 9). For each participant, all practice trials are projected onto the wall plane (the black trace represents trail 1, the white trace is trial 42). The time-series graphs show the pre- and post-test of the side-on condition. Shown are the time-normalized hip-wall angle (primary y-axis, blue line) and the time-series of the height of the hip position (secondary y-axis, red line). The histogram graphs show the pre- and post-test of the side-on condition. Shown is the relative time spent in different body-wall angles while mobile (black bins) or immobile (white bins) angle in degrees. Imm, Immobile; Mob, Mobile; t, total time.

In Figure 11 the individual results for participants 14 and 18 (along with participant 17) are presented. In contrast to the progressive improvement group, the nature of the histograms for this group are qualitatively different. For these individuals, in the pre-test, the histograms are less spread out and they are more concentrated toward a 0° value suggesting that while climbing, they were more face-on to the wall. The time-normalized data of the hip (primary axis of the line plots) provide support for this interpretation. Additionally, in clear contrast to the progressive improvement subgroup, the time series data of the hip reflect a general inability of these individuals to switch from a facing (~0°) and remain for any extended period of time in an oblique (around ±50°) or side-on (around ±90°) position relative to the wall. Also notable is that in the pre-test, participant 14 fell about halfway up the route (see the secondary red axis of the time-series data) and, that participant 18 took much longer to finish the route (355 s) than the participants in the progressive improvement subgroup (where the longest time for the progressive improvement group was 190 s). Finally, participant 17 was also included with this subgroup, because after examining her pre-test data, her hip-wall orientation was also concentrated around 0°. This leads us to speculate that if participant 17 had continued to practice, a sudden transition would have occurred in her performance dynamics.

#### No improvement

**Figure 12:**
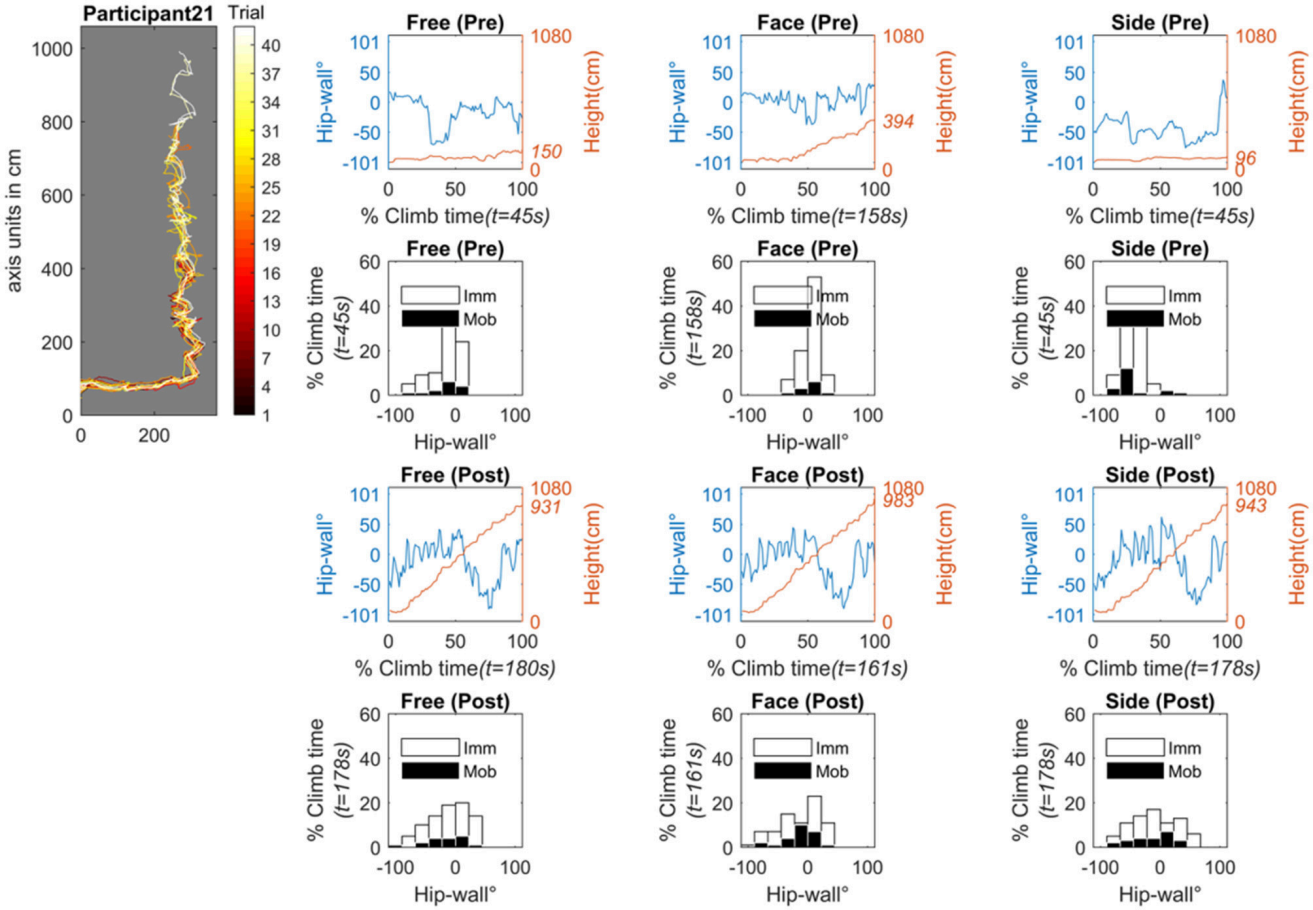
The individual (participant 21) who showed no improvement in terms of jerk, however, it should be clear that she did improve in other ways with practice. The graph on the top-left shows all practice trials projected onto the wall plane (the black trace represents trail 1, the white trace is trial 42). The pre-test data for the scanning procedure is shown in the top two rows of graphs and the bottom two rows show the post test data. The free condition (where the climber climbed as they liked) is given in the left column. The face condition (where the climber is asked to climb as much as possible while facing the wall) is given in the middle column. The side condition (where the climber is asked to climb as much as possible with the side of their body facing the wall) is given in the right column. Time series data are the time-normalized hip-wall angle (primary y-axis, blue line) and the time-series of the height of the hip position (secondary y-axis, red line). Histogram graphs show the pre- and post-test of the side-on condition. Shown is the relative time spent in different body-wall angles while mobile (black bins) or immobile (white bins) angle in degrees. Imm, Immobile; Mob, Mobile; t, total time.

Figure 12 shows the data for participant 21 (the individual showing “no improvement” in terms of jerk). Figure 12 shows all data from the pre- and post-test scanning procedure (free, face-on, and side-on). In the pre-test, across all conditions, participant 21 fell very early in the route. In the free and side-on condition she fell during the traverse (the first horizontal portion of the route). In the face-on condition, she was able to climb with a total vertical displacement of roughly 300 m (falling at about 400 m up the route). to be in a These findings indicate that the key difference between this individual in the pre-test and those in the progressive improvement and sudden improvement groups, was the ability to move vertically. Participant 21 appeared in a stage of learning where only postural stability was possible (i.e., traversing left and right, or standing stationary). Further examination her hip position data through practice also indicated that a significant amount of practice was required for her to successfully ascend the route. Interestingly, her learning curve in terms of jerk (Figure 8) shows a tendency to increase before finally, at around trial 27, it started to improve.

### Discussion of the individual analysis

In sum, the idea that some participants exhibited a specific learning dynamic as a function of their initial capabilities to ascend the route in a side-on position and move vertically, is a possible interpretation of these data (summarized in Figures 7, 8).

That is, it might be predicted that:

1. Individuals with a flat distribution of body wall orientation when required to be mobile whilst side-on will exhibit a progressive improvement in learning dynamics.
2. Individuals who exhibit a concentration toward a face-on position and show a limited capability to use a side-on coordination pattern will exhibit learning dynamics where an initial period of little improvement gives way to a sudden jump in performance.
3. Individuals unable to move vertically whilst face-on to the wall will exhibit very slow learning dynamics.

## General discussion

The aim of this study was to determine if through practice, individuals would acquire a new, more advanced pattern of coordination. However, this seemed only to occur for some participants and not others. Our exploratory findings revealed the nature of each individual's learning is not dependent on whether he/she can be classified prior to practice as a beginner (e.g., such as based on their amount of prior specific task experience or categorical ability level; Draper et al., 2011b). Rather, the nature of learning dynamics was likely dependent on each individual's behavioral repertoire prior to practice.

These findings lend support to previous work showing that individuals display different responses during learning to a given set of constraints, in terms of the nature and/or rate of learning (Liu et al., 2006, 2012). In Liu et al. (2006) participants were required to practice a hand held ball roller task over 7 days, where the aim was to achieve and maintain a certain rotation speed of the ball. In their study 3 out of 11 participants were not able to succeed in achieving a set criterion level of performance over the time given to practice. Additionally the successful participants had two subgroups of response: one subgroup improved in terms of both a qualitative and quantitative change and; the other subgroup did not undergo a transition (qualitative change) but still improved performance with practice. Other studies have also shown similar results, such that some individuals do not improve, others improve suddenly, and others improve gradually if at all (Vereijken et al., 1992, 1997; Delignières et al., 1998, 1999; Nourrit et al., 2000, 2003; Teulier et al., 2006; Teulier and Delignières, 2007). Although these studies have successfully identified different coordination regimes, they have largely failed to provide an understanding for why individuals differ in terms of their learning dynamics.

By identifying prior to practice current coordinative capabilities, our findings provide support for the idea that individual differences present prior to practice manifest themselves during practice in the rate and nature of learning. In doing so, differences in learning dynamics are explained as a function of the individuals prior repertoire of coordination (Kostrubiec et al., 2006, 2012). Indeed, in bi-manual coordination tasks (learning to finger waggle at specific frequencies and relative phase) where scanning procedures were initially operationalized (Zanone and Kelso, 1992), it has been proposed that there are two basic mechanisms or routes for learning a new required movement pattern–smooth shift or abrupt qualitative change (Kostrubiec et al., 2006, 2012). In cases where prior to learning, individuals who were initially bi-stable (able to produce in-phase and antiphase regimes), the tendency is for abrupt qualitative change in overall movement behavior. In individuals with initially multi-stable [able to produce in-phase, antiphase and some other regime(s)] solutions, the tendency is for a smooth shift in overall behavior.

Seifert et al. (2015) showed that experienced climbers tend to use more oblique positions of the hip relative to the wall when using climbing holds that encourage a side-on pattern of coordination. Similarly, in Orth et al. (2018), it was found that inexperienced climbers used more complex climbing trajectories on routes where holds encourage the use of side-on body positions. Furthermore, the beginners used less complex climbing trajectories when holds encouraged a face-on position. In contrast, a group of experienced climbers showed no significant differences in movement complexity across routes encouraging either face on and side-on climbing actions (Orth et al., 2018). In explaining the results of these studies (Seifert et al., 2015; Orth et al., 2018), the experienced climbers were deemed to have a larger repertoire of movement patterns that they could adapt as constraints (climbing hold orientation) changed. Beginners on the other hand, still needed to “find” these stable regimes of coordination. To explain the emergence of new movement patterns, it has been previously argued that exploration of different ways of grasping or using holds is a key mechanism (Seifert et al., 2013).

This study uncovered a more nuanced explanation, that the individuals behavioral repertoire is a key candidate for determining how exploration is functional, such as for either finding an efficient pathway through the route or finding new movement patterns of coordination (Orth et al., 2017a). This is an important distinction, since previous research in climbing has quantified exploration where the hand or foot comes into contact with a hold and is subsequently withdrawn without using that hold for progression or support (Pijpers et al., 2006; Orth et al., 2018). A key question in understanding the role of movement variability that accompanies the learning process is to determine the specific intentions that underlies exploratory behaviors (Orth et al., 2017b). According to the results in this study, we predict that a learners intentions will be in some way determined by their pre-existing repertoire of coordination. The way an individual will explore and learn in a given task is influenced by the number of movement solutions that they can already exhibit under that set of constraints (Kostrubiec et al., 2012). The implications for climbing in extreme environments is that climbing walls can be used as effective learning contexts for the development of movement skills in extreme environments, if accompanied by opportunities that enhance environmental knowledge and personal judgment skills.

More broadly, these findings suggest that, if the individual is able to safely explore (e.g., explore without failing the task), new movement patterns of coordination may be learned more rapidly (Seifert et al., 2015; Orth et al., 2018). Alternatively, in cases where a continuous improvement in performance occurs through practice, it is possible that these individuals do not need to qualitatively reorganize their overall movement patterning (Newell, 1985). Continuous improvement could reflect a refinement of a stable performance solution to achieve an outcome (Chow et al., 2007, 2008a). In this case, individuals may be able to effectively improve performance through making minor movement adaptations. In sum, these findings suggest when preparing individuals to learn in new contexts (for example, using the climbing wall environment to learn skills potentially required for extreme climbing environments), by understanding an individual's capabilities prior to practice, practitioners can more effectively plan the design of learning problems so that the individual is invited to seek out and potentially discover new motor solutions.

## Conclusion

In sum, personal (behavioral repertoire) and task/environmental (equipment, surfaces, edges, etc.) constraints that can influence effective exploration of a learning environment are an important consideration since they may influence whether or when a transition to a new movement solution eventuates (Delignières et al., 1998; Pacheco et al., 2017). In experimental designs where the search for new and functional solutions are allowed to emerge spontaneously (a solution is not proscribed), learners can display different trajectory dynamics (or learning curves). For example, different learning curves can include continuous improvement, sudden improvement; and, no improvement (Liu et al., 2006; Pacheco et al., 2017). One explanation for different routes to learning is the level of competition of a to-be-learned pattern with an already established behavioral repertoire (Nourrit et al., 2003; Kostrubiec et al., 2012).

A continuous-improvement in performance through practice may be more likely in individuals who do not need to dramatically modify their overall movement patterning and could reflect a refining of the current movement pattern to achieve the outcome. These individuals, may be able to effectively improve performance through making minor adjustments in control processes because their current behavioral repertoire is sufficient (Newell, 1985; Chow et al., 2008a). Alternately, individuals who exhibit sudden improvement can show higher levels of behavioral variability surrounding transitional periods which suggests the to-be-learned behavior is initially unstable (Teulier et al., 2006; Delignières et al., 2011). Finally, in situations where an individual does not improve through practice, the task dynamics may be too complex relative to the individual's current performance capabilities. A transitional (new) behavior may not surface, possibly preventing the individual from achieving the task goal even after extensive practice (Delignières et al., 1998; Liu et al., 2006; Pacheco et al., 2017). One reason individuals may show no improvement is that they do not have sufficient capability to explore effectively. The ability to explore, or exploration itself, has been identified as a candidate cause of sudden improvement as it may uncover “transitional information” needed to support a new mode of coordination (Newell, 1991; Teulier et al., 2006; Pacheco et al., 2017). In climbing, because of the added element of height from the ground and risk of injury due to falling, facilitating safe exploration is particularly relevant (Seifert et al., 2015). Indeed, if an individual feels unsafe to climb they can become more restricted in their movements (Pijpers et al., 2006), perhaps leading to ineffective exploration of the task dynamics.

One of the key challenges to the practitioner is to appropriately scale task difficulty relative to the learner over time. The data presented in this study suggests that task difficulty can be understood in terms of the extent to which the individual's current capabilities will compete or cooperate with the task. The level of competition may be better understood through operationalizing scanning procedures as exemplified in this study. Subsequently, the learner or coach can identify constraints that influence the individual's stability in the search of ways for achieving a fluent and successful climb on new routes. Performing in extreme environments requires effective decision making skills as well as climbing skills paying attention to individual differences in skill acquisition is important to ensure that learners undertake climbing in extreme environments at an appropriate time. Recognizing that individuals develop at different rates and in different ways depending on prior capacities not only supports effective skill acquisition in climbing but also broader preparation for climbing in extreme environments.

## Author contributions

DO, KD, LS: Planning experiments, developing rationale; DO: Performing data acquisition and analysis, and writing the manuscript; DO, KD, J-YC, EB, and LS: Revising follow up versions of the manuscript.

### Conflict of interest statement

The authors declare that the research was conducted in the absence of any commercial or financial relationships that could be construed as a potential conflict of interest.

## References

[B1] Billat V. Palleja P. Charlaix T. Rizzardo P. Janel N. (1995). Energy specificity of rock climbing and aerobic capacity in competitive sport rock climbers. J. Sports Med. Phys. Fitness 35, 20–24 7474988

[B2] Boulanger J. Seifert L. Hérault R. Coeurjolly J. F. (2016). Automatic sensor-based detection and classification of climbing activities. Sens. J. IEEE 16, 742–749. 10.1109/JSEN.2015.2481511

[B3] Bril B. Smaers J. Steele J. Rein R. Nonaka T. Dietrich G.. (2012). Functional mastery of percussive technology in nut-cracking and stone-flaking actions: experimental comparison and implications for the evolution of the human brain. Philos. Trans. R. Soc. BBiol. Sci. 367, 59–74. 10.1098/rstb.2011.0147 22106427 PMC3223788

[B4] Bruineberg J. Rietveld E. (2014). Self-organization, free energy minimization, and optimal grip on a field of affordances. Front. Hum. Neurosci. 8:599. 10.3389/fnhum.2014.00599 25161615 PMC4130179

[B5] Brymer E. Schweitzer R. (2013). The search for freedom in extreme sports: a phenomenological exploration. Psychol. Sport Exerc. 14, 865–873. 10.1016/j.psychsport.2013.07.004

[B6] Brymer E. Schweitzer R. (2017). Phenomenology and the Extreme Sports Experience. London: Routledge.

[B7] Chow J. Y. Davids K. Button C. Koh M. (2007). Variation in coordination of a discrete multiarticular action as a function of skill level. J. Mot. Behav. 39, 463–479. 10.3200/JMBR.39.6.463-480 18055353

[B8] Chow J. Y. Davids K. Button C. Koh M. (2008a). Coordination changes in a discrete multi-articular action as a function of practice. Acta Psychol. 127, 163–176. 10.1016/j.actpsy.2007.04.002 17555698

[B9] Chow J. Y. Davids K. Button C. Rein R. (2008b). Dynamics of movement patterning in learning a discrete multiarticular action. Mot. Control 12, 219–240. 10.1123/mcj.12.3.219 18698107

[B10] Chow J. Y. Davids K. Hristovski R. Araújo D. Passos P. (2011). Nonlinear pedagogy: learning design for self-organizing neurobiological systems. New Ideas Psychol. 29, 189–200. 10.1016/j.newideapsych.2010.10.001

[B11] Cordier P. Dietrich G. Pailhous J. (1996). Harmonic analysis of a complex motor behavior. Hum. Mov. Sci. 15, 789–807.

[B12] Cordier P. Mendès-France M. Bolon P. Pailhous J. (1994a). Thermodynamic study of motor behaviour optimization. Acta Biotheor. 42, 187–201.

[B13] Cordier P. Mendès-France M. Pailhous J. Bolon P. (1994b). Entropy as a global variable of the learning process. Hum. Mov. Sci. 13, 745–763.

[B14] Croft J. L. Pepping G. J. Button C. Chow J. Y. (2018). Children's perception of action boundaries and how it affects their climbing behavior. J. Exp. Child Psychol. 166, 134–146. 10.1016/j.jecp.2017.07.012 28888193

[B15] Davids K. Araújo D. Seifert L. Orth D. (2015). Expert Performance In Sport: An Ecological Dynamics Perspective, in Routledge Handbook of Sport Expertise, eds Baker J. Farrow D. (London: Routledge), 130–144.

[B16] Davids K. Button C. Bennett S. (2008). Dynamics of Skill Acquisition. A Constraints-Led Approach. Champaign, IL: Human Kinetics.

[B17] Delignières D. Marmelat V. Torre K. (2011). Degeneracy and long-range correlation: A simulation study, in Paper Presented at the BIO Web of Conferences (Montpellier).

[B18] Delignières D. Nourrit D. Deschamps T. Lauriot B. Caillou N. (1999). Effects of practice and task constraints on stiffness and friction functions in biological movements. Hum. Mov. Sci. 18, 769–793.

[B19] Delignières D. Nourrit D. Sioud R. Leroyer P. Zattara M. Micaleff J. P. (1998). Preferred coordination modes in the first steps of the learning of a complex gymnastics skill. Hum. Mov. Sci. 17, 221–241.

[B20] Draper N. Canalejo J. C. Fryer S. Dickson T. Winter D. Ellis G. (2011a). Reporting climbing grades and grouping categories for rock climbing. Isokinet. Exerc. Sci. 19, 273–280. 10.3233/IES-2011-0424

[B21] Draper N. Dickson T. Blackwell G. Fryer S. Priestley S. Winter D.. (2011b). Self-reported ability assessment in rock climbing. J. Sports Sci. 29, 851–858. 10.1080/02640414.2011.565362 21491325

[B22] Edelman G. M. Gally J. A. (2001). Degeneracy and complexity in biological systems. Proc. Natl. Acad. Sci. U.S.A. 98, 13763–13768. 10.1073/pnas.231499798 11698650 PMC61115

[B23] Fajen B. R. (2007). Affordance-based control of visually guided action. Ecol. Psychol. 19, 383–410. 10.1080/10407410701557877

[B24] Field A. (2009). Discovering statistics using SPSS, 3rd Edn London: Sage.

[B25] Fryer S. Dickson T. Draper N. Eltom M. Stoner L. Blackwell G. (2012). The effect of technique and ability on the VO_2_-heart rate relationship in rock climbing. Sports Technol. 5, 143–150. 10.1080/19346182.2012.755538

[B26] Fuss F. K. Weizman Y. Burr L. Niegl G. (2013). Assessment of grip difficulty of a smart climbing hold with increasing slope and decreasing depth. Sports Technol. 6, 122–129. 10.1080/19346182.2013.854800

[B27] Gibson J. J. (1979). The Ecological Approach to Visual Perception. Boston, MA: Houghton Mifflin.

[B28] Hristovski R. Davids K. Araújo D. Passos P. (2011). Constraints-induced emergence of functional novelty in complex neurobiological systems: a basis for creativity in sport. Nonlinear Dynamics Psychol. Life Sci. 15, 175–206. 21382260

[B29] Immonen T. Brymer E. Orth D. Davids K. Feletti F. Liukkonen J.. (2017). Understanding action and adventure sports participation—an ecological dynamics perspective. Sports Med. Open 3, 1–7. 10.1186/s40798-017-0084-1 28447331 PMC5406377

[B30] Kelso J. A. S. (2012). Multistability and metastability: understanding dynamic coordination in the brain. Philos. Trans. R. Soc. Lond. B. Biol. Sci. 376, 906–918. 10.1098/rstb.2011.0351 PMC3282307 22371613

[B31] Kostrubiec V. Tallet J. Zanone P. G. (2006). How a new behavioral pattern is stabilized with learning determines its persistence and flexibility in memory. Exp. Brain Res. 170, 238–244. 10.1007/s00221-005-0208-6 16328276

[B32] Kostrubiec V. Zanone P. G. Fuchs A. Kelso J. A. S. (2012). Beyond the blank slate: routes to learning new coordination patterns depend on the intrinsic dynamics of the learner: experimental evidence and theoretical model. Front. Hum. Neurosci. 6:222. 10.3389/fnhum.2012.00222 22876227 PMC3411071

[B33] Lack D. A. Sheet A. L. Entin J. M. Christenson D. C. (2012). Rock climbing rescues: causes, injuries, and trends in boulder county Colorado. Wilderness Environ. Med. 23, 223–230. 10.1016/j.wem.2012.04.002 22727678

[B34] Liu Y. T. Luo Z. Y. Mayer-Kress G. Newell K. M. (2012). Self-organized criticality and learning a new coordination task. Hum. Mov. Sci. 31, 40–54. 10.1016/j.humov.2011.06.005 21831466

[B35] Liu Y. T. Mayer-Kress G. Newell K. M. (2006). Qualitative and quantitative change in the dynamics of motor learning. J. Exp. Psychol. Hum. Percept. Perform. 32, 380–393. 10.1037/0096-1523.32.2.380 16634677

[B36] Llewellyn D. Sanchez X. Asghar A. Jones G. (2008). Self-efficacy, risk taking and performance in rock climbing. Pers. Individ. Dif. 45, 75–81. 10.1016/j.paid.2008.03.001

[B37] Madgwick S. O. H. Harrison A. J. L. Vaidyanathan A. (2011). Estimation Of Imu And Marg Orientation Using A Gradient Descent Algorithm, in Paper presented at the International Conference on Rehabilitation Robotics (Zurich). 10.1109/ICORR.2011.5975346 22275550

[B38] Newell K. M. (1985). Coordination, control and skill. Adv. Psychol. 27, 295–317.

[B39] Newell K. M. (1991). Motor skill acquisition. Annu. Rev. Psychol. 42, 213–237. 2018394 10.1146/annurev.ps.42.020191.001241

[B40] Nourrit D. Delignières D. Caillou N. Deschamps T. Lauriot B. (2003). On discontinuities in motor learning: a longitudinal study of complex skill acquisition on a ski-simulator. J. Mot. Behav. 35, 151–170. 10.1080/00222890309602130 12711586

[B41] Nourrit D. Deschamps T. Lauriot B. Caillou N. Delignieres D. (2000). The effects of required amplitude and practice on frequency stability and efficiency in a cyclical task. J. Sports Sci. 18, 201–212. 10.1080/026404100365108 10737271

[B42] Orth D. Button C. Davids K. Seifert L. (2017a). What current research tells us about skill acquisition in climbing, in The Science of Climbing and Mountaineering, eds Seifert L. Wolf P. Schweizer A. (Oxen: Routledge), 196–209.

[B43] Orth D. Davids K. Seifert L. (2018). Constraints representing a meta-stable régime facilitate exploration during practice and transfer of learning in a complex multi-articular task. Hum. Mov. Sci. 57, 291–302. 10.1016/j.humov.2017.09.007 28923581

[B44] Orth D. Kerr G. Davids K. Seifert L. (2017b). Analysis of relations between spatiotemporal movement regulation and performance of discrete actions reveals functionality in skilled climbing. Front. Psychol. 8:1744 10.3389/fpsyg.2017.01744 29056919 PMC5635808

[B45] Orth D. van der Kamp J. Memmert D. Savelsbergh G. (2017c). Creative motor actions as emerging from movement variability. Front. Psychol. 8:1903 10.3389/fpsyg.2017.01903 29163284 PMC5671646

[B46] Pacheco M. M. Hsieh T. Y. Newell K. M. (2017). Search strategies in practice: movement variability affords perception of task dynamics. Ecol. Psychol. 29, 243–258. 10.1080/10407413.2017.1368354

[B47] Pijpers J. R. Oudejans R. R. Bakker F. C. Beek P. J. (2006). The role of anxiety in perceiving and realizing affordances. Ecol. Psychol. 18, 131–161. 10.1207/s15326969eco1803_1

[B48] Prieske B. Withagen R. Smith J. Zaal F. T. (2015). Affordances in a simple playscape: are children attracted to challenging affordances? J. Environ. Psychol. 41, 101–111. 10.1016/j.jenvp.2014.11.011

[B49] Rietveld E. Kiverstein J. (2014). A rich landscape of affordances. Ecol. Psychol. 26, 325–352. 10.1080/10407413.2014.958035

[B50] Schöllhorn W. I. Mayer-Kress G. Newell K. M. Michelbrink M. (2009). Time scales of adaptive behavior and motor learning in the presence of stochastic perturbations. Hum. Mov. Sci. 28, 319–333. 10.1016/j.humov.2008.10.005 19062119

[B51] Schöner G. Zanone P. G. Kelso J. A. S. (1992). learning as change of coordination dynamics: theory and experiment. J. Mot. Behav. 24, 29–48. 14766496 10.1080/00222895.1992.9941599

[B52] Seifert L. Boulanger J. Orth D. Davids K. (2015). Environmental design shapes perceptual-motor exploration, learning, and transfer in climbing. Front. Psychol. 6:1819 10.3389/fpsyg.2015.01819 26635707 PMC4658451

[B53] Seifert L. Orth D. Boulanger J. Dovgalecs V. Hérault R. Davids K. (2014). Climbing skill and complexity of climbing wall design: assessment of jerk as a novel indicator of performance fluency. J. Appl. Biomech. 30, 619–625. 10.1123/jab.2014-0052 25010435

[B54] Seifert L. Orth D. Button C. Brymer E. Davids K. (2017). An ecological dynamics framework for the acquisition of perceptual–motor skills in climbing, in Extreme Sports Medicine, ed Feletti F. (Springer International Publishing), 365–382.

[B55] Seifert L. Orth D. Herault R. Davids K. (2013). Affordances and grasping action variability during rock climbing, in Studies in Perception and Action: Seventeenth International Conference on Perception and Action, eds Davis T. J. Passos P. Dicks M. Weast-Knapp J. A. (New York, NY: Psychology Press), 114–118.

[B56] Silva P. Garganta J. Araújo D. Davids K. Aguiar P. (2013). Shared knowledge or shared affordances? insights from an ecological dynamics approach to team coordination in sports. Sports Med. 43, 765–772. 10.1007/s40279-013-0070-9 23794235

[B57] Sumpter D. J. T. (2006). The principles of collective animal behaviour. Philos. Trans. R. Soc. B Biol. Sci. 361, 5–22. 10.1098/rstb.2005.1733 16553306 PMC1626537

[B58] Teulier C. Delignières D. (2007). The nature of the transition between novice and skilled coordination during learning to swing. Hum. Mov. Sci. 26, 376–392. 10.1016/j.humov.2007.01.013 17467091

[B59] Teulier C. Nourrit D. Delignières D. (2006). The evolution of oscillatory behavior during learning on a ski simulator. Res. Q. Exerc. Sport 77, 464–475. 10.1080/02701367.2006.10599381 17243221

[B60] Van Orden G. C. Kloos H. Wallot S. (2011). Living in the pink: Intentionality, wellbeing, and complexity, in Handbook of the Philosophy of Science, Vol. 10, ed Hooker C., *Philosophy of Complex Systems* (Amsterdam: Elsevier), 639–682.

[B61] Vereijken B. Van Emmerik R. E. A. Bongaardt R. Beek W. J. Newell K. M. (1997). Changing coordinative structures in complex skill acquisition. Hum. Mov. Sci. 16, 823–844.

[B62] Vereijken B. van Emmerik R. E. A. Whiting H. T. A. Newell K. M. (1992). Free(z)ing degrees of freedom in skill acquisition. J. Mot. Behav. 24, 133–142.

[B63] White D. J. Olsen P. D. (2010). A time motion analysis of bouldering style competitive rock climbing. J. Strength Cond. Res. 24, 1356–1360. 10.1519/JSC.0b013e3181cf75bd 20386481

[B64] Withagen R. De Poel H. J. Araújo D. Pepping G. J. (2012). Affordances can invite behavior: reconsidering the relationship between affordances and agency. New Ideas Psychol. 30, 250–258. 10.1016/j.newideapsych.2011.12.003

[B65] Zanone P. G. Kelso J. A. S. (1992). Evolution of behavioral attractors with learning: nonequilibrium phase transitions. J. Exp. Psychol. Hum. Percept. Perform. 18, 403–421. 1593227 10.1037//0096-1523.18.2.403

